# Evidence-based prediction and prevention of cardiovascular morbidity in adults treated for cancer

**DOI:** 10.1186/s40959-021-00105-y

**Published:** 2021-05-28

**Authors:** Renske Altena, Laila Hubbert, Narsis A. Kiani, Yvonne Wengström, Jonas Bergh, Elham Hedayati

**Affiliations:** 1 Department of Oncology and Pathology Cancer Center Karolinska, Karolinska Institutet, Stockholm, Sweden; 2 Medical Unit breast, endocrine tumours and sarcoma, Theme Cancer, Karolinska University Hospital Stockholm, Solna, Sweden; 3 Department of Cardiology and Department of Health, Medicine and Caring Sciences, Linköping University, Norrköping, Sweden; 4 Department of Neurobiology, Care Sciences and Society, Division of Nursing, Karolinska Institutet, Stockholm, Sweden

**Keywords:** Prevention, Cancer survivor, Evidence-based, Cardiotoxicity, Lifestyle, Exercise

## Abstract

**Background:**

Cancer treatment-related morbidity relevantly compromises health status in cancer survivors, and efforts to optimise health-related outcomes in this population are vital to maximising healthy survivorship. A pre-treatment assessment – and possibly preventive management strategies – of cancer patients at increased risk for cardiovascular disease (CVD) seems a rational approach in this regard. Definitive evidence for such strategies is largely lacking, thereby impeding the formulation of firm recommendations.

**Results:**

The current scoping review aims to summarise and grade the evidence regarding strategies for prediction and prevention of CVD in adults in relation to oncological treatments. We conducted a scoping literature search for different strategies for primary prevention, such as medical and lifestyle interventions, as well as the use of predictive risk scores. We identified studies with moderate to good strength and up to now limited evidence to recommend primary preventive strategies in unselected patients treated with potentially cardiotoxic oncologic therapies.

**Conclusion:**

Efforts to minimize the CVD burden in cancer survivors are needed to accomplish healthy survivorship. This can be done by means of robust models predictive for CVD events or application of interventions during or after oncological treatments. Up to now there is insufficient evidence to implement preventive strategies in an unselected group of patients treated with potential cardiotoxic oncological treatments. We conclude that randomised controlled trials are needed that evaluate medical and lifestyle interventions in groups at increased risk for complications, in order to be able to influence chronic illness risks, such as cardiovascular complications, for cancer survivors.

## Highlights

- Minimising treatment-related cardiovascular disease (CVD) in cancer survivors is crucial to maximising healthy survivorship.
- Predictive models that aid in identifying persons at highest risk for such toxicities as well as primary prevention may be approached to decrease CVD after cancer treatments.
- We performed a scoping literature review on prediction and primary prevention of CVD in cancer survivors and graded the studies with a Level of Evidence.
- We identified studies with moderate to good strength and up to now limited evidence to recommend primary preventive strategies in unselected patients treated with potentially cardiotoxic oncologic therapies. Some predictive models exist, but those lack methodological robustness to be implemented in clinical use.

## Background

Health prospects after a cancer diagnosis have improved impressively over the past decades. There is a steadily improving life-expectancy for patients with cancer, with an age-standardised 10-year survival of 70% for patients diagnosed with breast cancer, 80% for Hodgkin’s disease, and 90% for testicular cancer in Western countries [1, 2]. Worldwide, the number of new cancer cases is rising and was estimated to be 18.1 million in 2018, leading to a large and growing group of cancer survivors [3, 4].

Several factors contribute to these improved cure rates, including earlier detection of some cancers due to screening strategies, the organisation of care in centres of expertise and more effective treatment regimens [3]. Relatively recently a trend towards de-escalation of (curative) oncologic treatments has begun, e.g. in breast cancer [5], meaning that studies are designed to reduce the amount and intensity of treatment patients receive while maintaining equally good cancer outcomes. However, still, many patients with early-stage malignancies will receive treatment combinations of surgery, radio-, chemo- and targeted therapies. Different treatment modalities have different modes of action, levels of effectiveness, as well as the scope of acute and late side-effects. Consequently, a large and growing group of cancer survivors is at risk to develop treatment-related toxicity. Long-term complications include the development of cardiovascular disease (CVD), sub−/infertility, neurotoxicity and second malignancies [6]. Efforts to reduce disease burden, both in terms of risk for disease relapse and long-term treatment-related toxicity, in this population are vital to maximise healthy survivorship and improve quality of life.

CVD can arise during oncological treatment, as well as up to decades after treatment completion. Significantly, not only direct treatment-induced toxicity can contribute to this development, but also (shared) risk-factors for both CVD and malignancy can increase the risk for this morbidity [7–13].

**Fig. 1:**
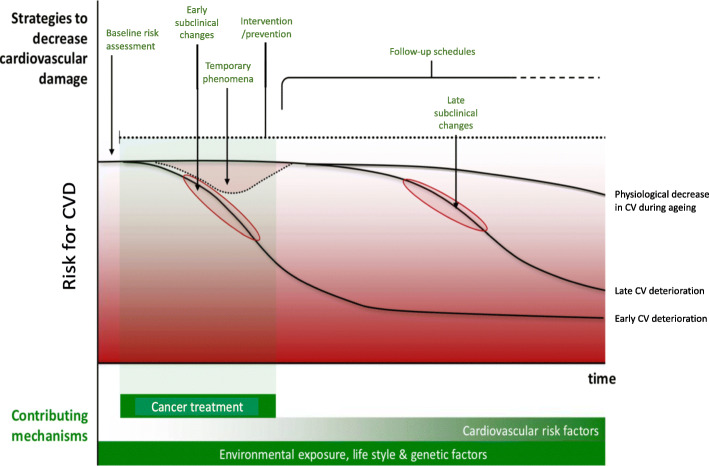
Timing of development of cancer treatment-related cardiovascular disease and time-points for primary prevention. Hypothetical schematic representation of the risk for development of CVD in people treated for malignancy! Adapted from Altena et al., Lancet Oncology 2009 [14]

To achieve an optimal health status for cancer survivors, detection and – when possible – prevention of these issues is of high priority. The development of CVD is a gradual process that progresses from subclinical changes to clinical morbidity, a process that may be accelerated by damage arising from oncological treatments (Fig. 1). Therefore, a baseline risk assessment followed by early interventions in case of therapies associated with a high risk for CVD morbidity as well as for persons at increased risk might be effective to impede or stop the progression towards overt CVD in cancer patients.

The current review aims to concisely summarise methods for baseline risk assessment, primary prevention with medication as well as lifestyle interventions in adult cancer patients who are receiving oncological treatments that may cause cardiovascular morbidity.

## Methods

**Fig. 2:**
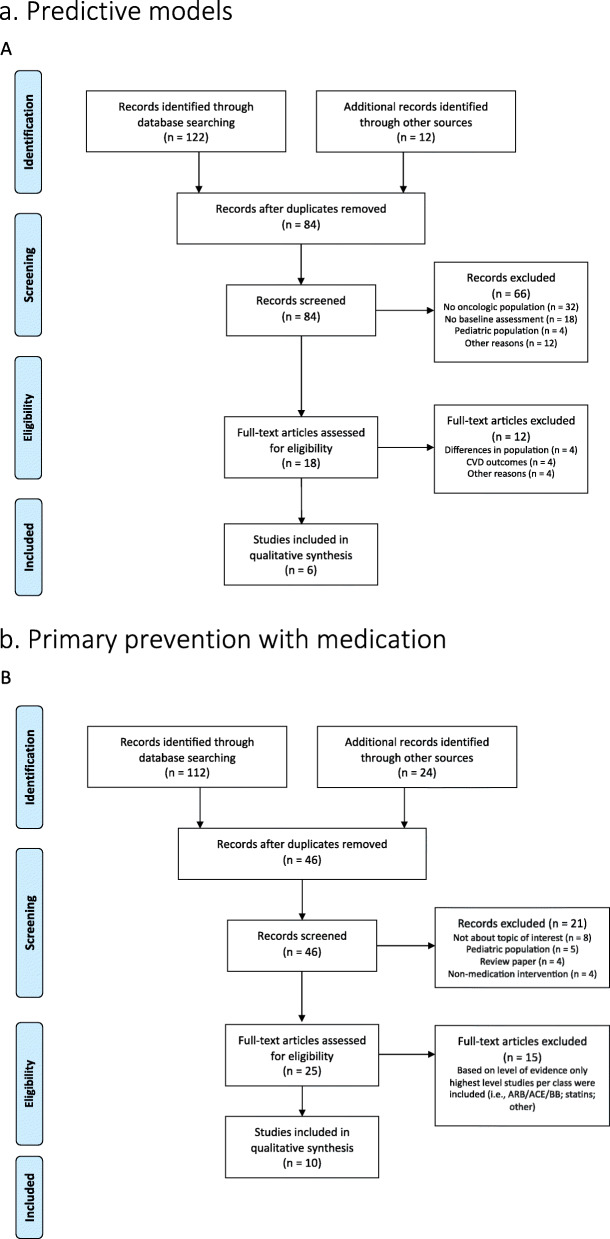
PRISMA flow-chart included trials. **a** Predictive models. **b** Primary prevention with medication

The literature was reviewed on the different topics covered in this article, such as baseline risk prediction tools, primary prevention with medication and lifestyle interventions. The PubMed and Cochrane databases were used for this purpose as well as ClinicalTrials.gov. Peer-reviewed papers published in medical journals on clinical trials in human adults and literature reviews, written in the English language between January 1990 – September 2020 were included. Figure 2 provides a PRISMA flow chart of the selection process and the included manuscripts for the topics prediction tools and primary prevention with medication. The available data for lifestyle interventions is too scarce to assign a level of evidence to, and therefore, we cover this part as a narrative section.

Each item is assigned a level of evidence (LOE) backing up its applicability, according to the system used by the Oxford Centre of Evidence-Based Medicine (Table 1). Level 1a evidence is viewed as definite, and level 5 as weak. To maintain a concise summary of the literature, we decided to only refer to papers within the highest level of evidence for each reviewed topic, summarised in Table 2.

**Table 1:** Levels of Evidence according to the Oxford Center of Evidence Based medicine (https://www.cebm.net/2009/06/oxford-centre-evidence-based-medicine-levels-evidence-march-2009/)

Level	1	a	Systematic review of randomised controlled trials
		b	Single randomised controlled trial
Level	2	a	Systematic review of cohort studies
		b	Individual cohort study
		c	“Outcomes” research
Level	3	a	Systematic review of Case-Control studies
		b	Individual Case-Control study
Level	4		Case-series
Level	5		Expert opinion

**Table 2:** Predictive models for cardiovascular events in cancer patients

Study	Population	Components of model	Discriminative value	Strength	Consecutive step
Ezaz et al. [15]	From SEER database: *N* = 1664 pts. treated for HER2+ BC with systemic therapies	1 point: past medical history of hypertension, diabetes, or age 75–79 years. 2 points: history of coronary artery disease, renal failure, or atrial fibrillation or flutter, having received any chemotherapy or > 80 years of age	Three risk groups: low (0 to 3 points), medium (4 to 5 points), and high (≥6 points) risk strata with 3-year CE rates of 16.2, 26.0, and 39.5%, respectively	Training set of 70%, internal validation in the other 30% with strong performance of the model	Validation in additional external cohorts. No information on cardiovascular medication use
Rushton et al. [16]	*N* = 143 patients with HER2+ BC referred to a cardio-oncology clinic at a tertiary care center	Sensitivity analysis to validate model composed by Ezaz et a [16]	Low risk: 42% CE rate, 13% permanent HF Moderate risk: 64% CE rate, 14% permanent HF High risk: 30% CE rate, 20% permanent HF	Low cardiac risk score had a negative predictive value of 94% for permanent cardiotoxicity.	Highly selected population. Sub-optimal performance in high risk group.
Fogarassy et al. [8]	Nationwide health care databases, *N* = 8068 BC pts. treated with epirubicin	Risk-prediction score for HF composed of age, diabetes mellitus, hypertension, coronary artery disease, stroke, epirubicin dose, docetaxel dose, capecitabine, gemcitabine, bevacizumab and cancer stage	Five score point categories and corresponding risk for HF; score 1–7 HF 2.1%, score 8–9 HF 5.0%, score 10–12 HF 10.3%, score 13–18 HF 22.1%, score 19–26 HF 31.7%	Large dataset, Training set of 70%, internal validation in the other 30%	Information on cardiovascular medication use. External validation.
Romond et al. [17]	Analysis from NSABP B-31 trial, *N* = 1830 pts. with HER2+ BC	Retrospective regression analysis to reveal predictors for cardiac events: formula to calculate cardiac risk score	Cardiac risk score based on age and baseline LVEF by MUGA	High discriminate ability (C-index 72%) in associating the length of time to a cardiac event with the probability of not experiencing CEs.	No external validation of the risk score
Hermann et al. [18]	Literature- and expert-based recommendation	Type of treatment, age, gender, history of CVD or presence of risk factors for CVD	No statistical validation	Easily accessible variables	Test algorithm in patient population for clinically relevant endpoints.
Abdel-Qadir et al. [19]	Real-world population EBC Ontario 2003–2015, *N* = 90,104 (2/3 training, 1/3 validation set)	Risk-prediction score for MACE composed of age, hypertension, diabetes, ischaemic heart disease, atrial fibrillation, HF, cerebrovascular disease, peripheral vascular disease, chronic obstructive pulmonary disease, and chronic kidney disease	Ten-year MACE incidence was > 40-fold higher for patients in the highest score decile compared to the lowest. The c-index was 81.9% (95% confidence interval 80.9–82.9%) at 5 years and 79.8% (78.8–80.8%) at 10 years in the validation cohort, with good agreement between predicted and observed MACE incidence.	Clinically relevant long-term outcome	No incorporation of cancer and treatment-related variables
Dranitsaris et al. [20]	Metastatic breast cancer pts. treated with anthracyclines (doxorubicin or liposomal doxorubicin), *N* = 509	Risk scoring algorithm (range 0–62) based on number of cumulative cycles, patient age and weight, previous anthracycline exposure and poor performance statu	A ROC analysis had an area under the curve (AUC) of 0.84 (95% CI: 0.79–0.89). A precycle risk score cutoff of ≥30 to < 40 was identified to optimally balance sensitivity (58.5%) and specificity (89.0%).	Easily accesible variables	Validation in external cohorts. Add information on risk factors for CVD and medication use .

*Abbreviations*: *HER2* Human Epidermal growth factor Receptor 2, *pts* patients, *RCT* randomised controlled trial, *yr* year, *LVEF* left ventricular ejection fraction, *CVD* cardiovascular disease, *HF* heart failure, *CE* cardiac event

## Review / main text

### Prediction of future CVD in cancer patients

An accurate and practically applicable model that predicts the baseline risk for development of future CVD is needed in patients with a newly diagnosed malignant disease scheduled for anti-cancer treatment (Fig. 1). Timely and accurate risk prediction is crucial to adapt treatment plans and initiate (preventive) CVD treatment to balance optimal cancer therapy whilst minimising the risks of development of CVD.

Up to date, it is not known whether predictive models for CVD that are used in the general population, e.g. the Framingham Risk Score, are representative of the future CVD risk in cancer survivors treated with therapies that are potentially harmful to the cardiovascular system. Theoretically, one could argue that such models are not entirely representative, as there are additional pathogenic factors that contribute to the development of CVD in the specific population (e.g. chemotherapy toxicity causing direct cardiomyocyte damage and accelerated atherosclerosis) [21]. A cross-sectional study among testicular cancer survivors revealed no differences in the risk score on the Framingham Risk Score when compared to the score of age-matched persons in the general population, 1 year after chemotherapy treatment [22]. However, long-term follow-up studies have confirmed the increased risk of late CVD in this population [23–25].

The recently published guideline from the European Society of Medical Oncology (ESMO) recommends a thorough baseline screening for CVD risk factors before an anti-cancer treatment is initiated, but does not include a discussion about the use of predictive models [26]. The guideline of the American Society of Clinical Oncology (ASCO) on prevention and monitoring of cardiotoxicity in adult cancer survivors provides an approach on how patients can be divided into different risk groups for future CVD, based on oncological treatment, age and presence of risk factors for CVD. This risk staging is though mainly evidence-based with intermediate-moderate quality evidence [27].

The only area in which such predictive models, based on clinical features and treatment factors, for future CVD have been investigated are breast cancer patients. To the best of our knowledge, seven studies have been published on this topic (Fig. 2, Table 2) [8, 15–20]. Table 2 summarises the respective study characteristics and the populations included in the analyses. The study by Ezaz et al. showed a clinically useful model where patients with HER2-positive breast cancer were divided into low, medium and high-risk groups for heart failure/cardiomyopathy, with relatively good discriminative ability [15]. This study used part of their cohort as an internal validation of the model, and later, Rushton et al. used this model in their data set of a very selected group of breast cancer patients referred to a tertiary care centre after they had developed cardiac evens in relation to cancer treatment [8]. The most important conclusion from the latter analysis was that the application of this cardiac risk score helped identify those at low risk of permanent cardiac dysfunction. However, it did not perform as well in identifying high-risk patients.

A similar approach was used in the analysis by Fogarassy et al., where a cohort of over 8000 breast cancer patients treated with epirubicin was analysed [8]. Here, risk factors for late heart failure were identified in a training set of 70% of this cohort and internally validated in the other 30%. The paper by Hermann et al. gives an expert-opinion based recommendation on how patients at high risk for future CVD can be identified, monitored and managed to decrease the risk for future CVD. Factors like the exact type of treatment, age, gender, history of CVD or presence of risk factors for CVD are included in their general Cardio-Oncology algorithm [18]. Abdel-Qadir et al. used a large cohort of breast cancer survivors treated in Ontario as a model for developing and validating a predictive model and composed a risk score for the future development of major cardiovascular events based on baseline factors such as age and presence of CVD [19]. This model had the good discriminative ability and was also consistent in the validation set, but no cancer treatment-related factors were included in this model. In addition, one study investigated a predictive risk score for development of cardiac toxicity from anthracyclines in patients with metastatic breast cancer [20].

In summary, three baseline predictive models for heart failure in breast cancer patients have been composed, and the one developed by Ezaz et al. in HER2-positive breast cancer patients [15] was validated in highly selected external cohort with moderate performance [16]. Our literature search did not identify comparable studies that have been performed in other cancer patient populations.

*Highest LOE for predictive models: 2b*.

### Prevention of future CVD during oncological treatments

Primary prevention refers to delaying or preventing the onset of CVD, whereas secondary prevention aims to reduce the number of new or severe cases of CVD. Table 3 summarises the currently published studies in this area, where we have only included papers with the highest level of evidence (according to Table 1) within a specific class of medication. The role of intervention with angiotensin-converting enzyme (ACEiI) inhibitors, angiotensin-receptor blockers (ARB), beta-blockers, statins, aspirin/anticoagulants and other drugs will be discussed, respectively.

**Table 3:** Summary of prospective randomised controlled trials on medical prevention of cardiac dysfunction during oncological treatment

Intervention (LOE)	Population	Study design	Primary outcome	Result
**ACEI/ARB/beta-blockers**	Meta-analysis *N* = 2301 early breast cancer pts., HER2+/− [28]	RCT’s ACEI, ARB and/or BB	Change in LVEF	Standardized mean difference LVEF baseline vs treatment completion for all groups: − 2.36 [95% CI: − 3.23 to − 1.49]
	Meta-analysis *N* = 633 pts. [29]	RCT’s carvedilol vs placebo	Occurrence of low LVEF	Low LVEF carvedilol vs. placebo 3.2% vs. 5.8% (OR: 0.42; 95% confidence interval: 0.18–0.99; *p* = 0.05). LVEF reduction carvedilol vs. placebo: mean difference 2.41% (95% CI: 0.01–4.81; *p* = 0.05).
	Meta-analysis *N* = 1984 pts. [30]	RCT’s with neurohormonal therapy vs placebo (BB, ACEi, ARB, mineralocorticoid receptor antagonists)	Change in LVEF	Intervention arms had a higher LVEF on follow-up (standardized mean difference + 1.04% (95% CI 0.57–1.50) but significant heterogeneity (I^2^ 96%)
	*N* = 69 pts. with malignancy treated with anthracycline [29]	Prospective RCT, single-blind, enalapril vs. placebo	Change in LVEF from baseline to 6 months	Preserved LVEF in enalapril group (*p* = 0.58), decreased LVEF in control group (*p* < 0.001)
	*N* = 40 pts. with non-Hodgkin lymphoma, combination (CHOP) chemotherapy [31]	Prospective RCT, open-label, valsartan (80 mg/day) vs. control	Explorative; changes in neurohumoral, echocardiographic, electrocardiographic markers during therapy	Compared to control, valsartan significantly inhibited the dilatation of LVDd (*P* = 0.01), elevation of BNP (*P* = 0.001), and prolongation of the QTc interval and QTc dispersion (*P* = 0.0009 and *P* = 0.02, respectively) after CHOP chemotherapy
	*N* = 49 pts., different malignancies treated with epirubicin [32]	Prospective RCT, telmisartan vs. placebo	Change in strain rate by echocardiography compared to baseline. Biomarkers.	Decrease in strain rate in both arms, but recovery to baseline values in telmisartan group. Lower levels of inflammatory marker IL-6 in telmisartan group compared to control. No differences in (changes in) LVEF.
	*N* = 36 acute leukemia receiving intensive chemotherapy, *N* = 54 hematologic malignancies receiving stem cell transplantation. LVEF ≥50% at baseline [33].	Prospective RCT, carvedilol + enalapril vs. control group	Change in LVEF by echocardiography	Preserved LVEF in intervention arm (− 3.28 [− 5.49 to − 1.07] in control group, − 0.17 [− 2.24 to 1.90] intervention group, difference − 3.11%, *p* = 0.04) In control group lower incidence death/heart failure (6.7% vs. 22%, *p* = 0.036) and of death, heart failure, or a final LVEF < 45% (6.7% vs. 24.4%, *p* = 0.02).
	*N* = 147 lymphoma pts., doxorubicin combination chemotherapy [34]	Prospective randomised three-arm trial (metoprolol, enalapril or placebo)	Clinical heart failure and subclinical cardiotoxicity	No statistically significant differences in the study arms (up to 10 yr follow-up) [35]
	*N* = 468 early HER2+ breast cancer pts., treated with trastuzumab +/− anthracyclines [36]	Prospective randomised three-arm trial (lisinopril, carvedilol or placebo)	Cardiotoxic event	Cardiotoxicity occurred in 32% patients on placebo, 29% on carvedilol, and 30% on lisinopril.
	*N* = 121 pts. with early breast cancer treated with adjuvant chemotherapy including anthracyclines [37]	Prospective 2 × 2 factorial placebo-controlled double-blind RCT.	Change in LVEF	The overall decline in LVEF was 2.6% (95% CI 1.5, 3.8) in the placebo group and 0.8 (95% CI − 0.4, 1.9) in the candesartan group in the intention-to-treat analysis (*P*-value for between-group difference: 0.026). No effect of metoprolol on the overall decline in LVEF was observed.
	*N* = 94 pts. with early HER2+ breast cancer [38]	Prospective randomised three-arm trial (perindopril, bisoprolol or placebo)	Trastuzumab-mediated left ventricular remodeling on ultrasound	No difference between groups
**Statins**	*N* = 40 pts. with malignancy planned for anthracycline treatment [39]	Prospective RCT 6 months of atorvastatin (40 mg/day) vs. control	Rate LVEF < 50%	No difference primary endpoint (*p* = 0.18), but mean change in LVEF in control group significantly larger than in statin group (− 7.9 ± 8.0 vs. 1.3 ± 3.8, *p* < 0.001)
**Other**	Meta-analysis of 2177 breast cancer pts. treated with anthracyclines +/− trastuzumab [40]	Dexrazoxane vs placebo	Rate of CHF and cardiac events	Reduction in CHF (RR: 0.19; 95% CI: 0.09 to 0.40, *P* < 0.001) and cardiac events (RR: 0.36; 95% CI: 0.27 to 0.49, *p* < 0.001)
	*N* = 27 pts. treated with doxorubicin [41]	Prospective open label RCT, sildanefil (100 mg 3 times daily) vs. control	Change in mean LVEF	No difference. No difference in (changes in) serum troponin I.

*Abbreviations*: *HER2* Human Epidermal growth factor Receptor 2, *pts* patients,*RCT* randomised controlled trial, *ACEI* Angiotensin Enzyme Converse Inhibitors, *ARB* Angiotensin Receptor Blockers, *BB* beta-blockers, *yr* year, *LVEF* left ventricular ejection fraction, *LVDd* left ventricular diastolic diameter, *BNP* brain natriuretic peptide, *IL-6* interleukin 6, *TDI* tissue Doppler imaging, *OR* odds ratios, *CI* confidence interval

Most intervention studies have been performed in patients treated with anthracyclines, mainly breast cancer patients. The majority of the studies investigated preventive measures during and up to shortly after completion of a potentially toxic therapy in unselected patients.

#### Preventive medication: (ACEI/ARB/beta-blockers)

Recently, a meta-analysis reported on the available evidence about primary prevention with renin-angiotensin-aldosterone inhibitors such as ACEI and ARB, and beta-blockers, in breast cancer patients treated with anthracyclines with and without trastuzumab [28]. A total of *N* = 2301 patients was included, and the cardioprotective effect of the different drugs was estimated. Median changes in LVEF at three time-points (immediate after completion of chemotherapy, at six- and 12-months post-treatment) and the incidence of heart failure (CHF), was statistically significantly lower in the intervention groups compared to the control. However, the magnitude of the effect was modest and the confidence intervals (CI) for the last two time-points rather broad (standardized mean difference in LVEF -chemotherapy completion − 2.36 [95% CI: − 3.23 to − 1.49]; 6 months − 6.54 [95% CI: − 10.74 to − 2.34]; 12 months − 5.37 [95% CI: − 9.31 to − 1.43]). In the three trials included in the meta-analysis that included patients with HER2-positive breast cancer treated with trastuzumab, no effect of the intervention was noted in terms of LVEF or rate of CHF. There was neither a persistent effect seen of beta-blockers in terms of one-year LVEF-outcomes in the entire group. The authors conclude that prophylactic treatment with ARB/ACEI and beta-blockers of unselected breast cancer patients scheduled for anthracycline +/− trastuzumab should not be routinely recommended. This finding was also noted in a large prospective study in unselected patients with HER2-positive breast cancer that were randomized to candesartan or placebo during and after adjuvant systemic therapy [29]. Contrarily, selecting high-risk patients that are candidates for interventions aimed to decrease rates of cardiotoxicity might be a rationale strategy, as was demonstrated in large Italian study [42]. Here, patients with rises in troponin shortly after high-dose chemotherapy for breast cancer hade lower rates of late cardiotoxicity (decreases in LVEF of > 10%) when they received treatment with enalapril. In line with these findings, we believe future studies should aim to identify high-risk patients and specifically target studies in such a population.

Of interest, three prospective randomized trials in early breast cancer patients have directly compared the role of ACEi/ARB versus beta-blockers to placebo [36–38]. Results from these trials, having somewhat different patient populations and primary endpoints, do not favour the use of either one of these classes of drugs (Table 3).

A meta-analysis that focused on beta-blockade included six trials (in total *N* = 633 patients) that investigated the role of carvedilol in primary prevention of anthracycline-induced cardiac toxicity. This study revealed a protective effect as apparent from reduced rates of pathological LVEF (defined as a LVEF < 50%) favouring the carvedilol group (3.2% vs 5.8%; odds ratio 0.42 [95% CI: 0.18 to 0.99]). Furthermore, there were significantly smaller reductions in LVEF in carvedilol-treated patients than in placebo-treated patients (mean differences: 2.41% [95% CI: 0.01 to 4.81; *p* = 0.05) [43]. The NNT of 38 implies that 38 patients have to be treated with carvedilol to prevent a LVEF-decrease to < 50% in one patient.

Small studies have been performed in patients with other non-breast cancers (Table 2); in a cohort of *N* = 40 non-Hodgkin lymphoma patients, treatment with the ARB valsartan resulted in ameliorated changes in subclinical parameters for cardiac toxicity (such as echocardiographic parameters and biomarkers), compared to controls who did not receive valsartan [31].

In the OVERCOME trial (NCT01110824), the combination of carvedilol plus enalapril resulted in preserved LVEF 6 months after chemotherapy treatment for hematologic malignancies, compared to a control group without this medication combination (*N* = 90) [33].

*Highest LOE for the use of ACEI/ARB: 1b, for beta-blockade: 1a (both: small protective effect on LVEF)*.

### Preventive medication: statins

Statins can lower the level of low-density lipoprotein (LDL) cholesterol in the blood. High levels of LDL cholesterol can lead to atherosclerosis and CVD. A small randomised controlled trial on atorvastatin 40 mg/daily for 6 months during anthracycline therapy for various malignancies demonstrated that the intervention group had a preserved LVEF, which was one of the secondary endpoints of the study, compared to a decline of almost 8% in LVEF in the control group [39]. There was no statistically significant difference in the primary endpoint of the trial, namely a LVEF < 50% after anthracycline treatment.

*Highest LOE for the use of statins: 1b (no difference in LVEF < 50%)*.

### Preventive medication: aspirin/anticoagulants

Acetylsalicylic acid (ASA, aspirin) inhibit platelet aggregation. Low dose ASA prevent myocardial infarction and stroke in high risk individuals. To our knowledge, no clinical studies have been conducted or are ongoing regarding the role of aspirin or anticoagulants in the primary prevention of cancer therapy-related cardiotoxicity. However, the preventive effect on the development of venous thrombosis during oncological treatments is established for high-risk groups [44, 45].

Some preclinical studies have reported on the role of prostacyclins in the development of anthracycline-induced cardiomyocyte damage, and therefore theoretically, cyclooxygenase 1 and 2 inhibitors could be effective in prevention cardiotoxicity [46, 47]. However, there are no available clinical studies with this class of drugs.

*Highest LOE for aspirin/anticoagulants: not available*.

### Preventive medication: dexrazoxane and sildenafil

The use of the iron-chelator dexrazoxane is mainly established in pediatric cancer patients as a drug for primary prevention of cardiotoxicity related to anthracycline therapy. In 2019, a systematic literature review on the use of dexrazoxane in adults was published [40]. This review identified seven trials in breast cancer patients, and the meta-analysis of these trials with in total 2177 breast cancer patients revealed a statistically significant and clinically relevant protective effect on the rate of CHF and cardiac events, without signs of impaired oncological treatment efficacy.

In a small prospective study in women treated with doxorubicin (NCT01375699), treatment with sildenafil (100 mg daily, 300 mg at treatment days) did not prevent declines in LVEF or rises in high sensitivity cardiac troponin I [41]. This drug is used very rarely in clinical practice, and the available evidence does not support use as of now.

*Highest LOE for other drugs – dexrazoxane: 1a (reduces risk of CHF and cardiac events), sildenafil: 1b (no difference LVEF-declines)*.

### Lifestyle interventions before and during treatment initiation

Recently, a survey among various medical professionals involved in the care of cancer survivors reported a high rate of health promotion mainly amongst primary care physicians, but a much lower rate among oncologists and other specialists [48]. Many reported lack of time as well as concerns with compliance among cancer survivors as reasons for abstaining health-promoting recommendations.

Several retrospective cohort studies have shown associations between lifestyle habits (such as dietary intake, physical activity, alcohol consumption and smoking status) and the risk for long-term cardiovascular morbidity and mortality after cancer therapy, e.g. in a cohort of Hodgkin lymphoma, hematopoietic cell transplantation and colorectal cancer survivors [49–51]. A retrospective analysis from the Women’s Health Initiative cohort showed that higher levels of self-reported physical activity were associated with a significant graded reduction in CV events in breast cancer survivors [52]. Various sources can introduce bias and Influence these findings, and a causal relationship has, therefore, not been established yet. To our knowledge, no prospective trials have been performed that investigate lifestyle interventions, such as physical exercise, smoking cessation and diet changes, concerning objective and clinically relevant cardiovascular endpoints.

The ASCO guidelines on breast cancer survivorship [53], as well as the ASCO and ESMO guidelines on prevention of cardiac dysfunction in cancer survivors [26, 27], underscore the importance of screening for and educating (breast) cancer survivors about abovementioned lifestyle modifications, that may reduce the risk for or severity of cardiotoxicity or cardiovascular diseases in this population. Associations, and to some extent also causality, between such modifications and health outcomes in the general population, is evident from several extensive studies.

#### Smoking cessation

A meta-analysis showed that smoking cessation rates after a cancer diagnosis are rather low, even after intervention programmes [54]. No prospective data are supporting the hypothesis that smoking cessation might improve health prospects after a cancer diagnosis, including a lower rate of CVD as well as improved oncologic outcomes. However, indirect evidence originates, for example, from a large meta-analysis that found evidence for an increased risk for radiotherapy-induced cardiac toxicity in smokers [13]. The current lack of evidence should, however, not discourage professionals from stimulating smoking cessation.

*Highest LOE for smoking cessation and CVD endpoints: n.a*.

### Diet changes and weight loss

Weight gain after a breast cancer diagnosis is common. Cespedes et al. reported a weight gain of > 4.5 kg/10 lbs. among 25% of a total of 2000 breast cancer patients [55]. However, this did not translate into increased long-term risk for CVD. A large prospective intervention study in early breast cancer patients on dietary changes, with increases in fruit and dietary fibre consumption and reductions in dietary fat intake, revealed no difference in breast cancer-related outcomes nor overall mortality [56]. A cohort study in breast cancer survivors showed that women who had a prudent (balanced) diet, consisting of high intakes of fruits, vegetables, whole grains, and poultry, had improved survival as well as a lower non-breast cancer-related survival, which may thus relate to a lower rate of death due to CVD [57]. On the contrary, a systematic review on the impact of dietary patterns in breast cancer and colorectal cancer survivors was not able to find any positive associations between dietary patterns and risk for disease recurrence and overall mortality [58]. However, the review did not specify CVD-related endpoints.

*Highest LOE for diet interventions/weight loss and CVD endpoints: n.a. (circumstantial: reduction non-BC mortality in BC survivors)*.

### Physical exercise

Most intervention studies on physical exercise interventions have been performed in early breast cancer patients, as well as in smaller populations of patients with colorectal and prostate cancer. Physical exercise interventions improve physical performance, cardiorespiratory fitness and fatigue during and shortly after breast cancer chemotherapy treatment completion [59, 60]. A systematic review and random-effects meta-analysis in almost 50,000 breast and colorectal cancer patients showed that patients who increased their physical activity by any level from pre- to post-diagnosis showed a total reduction in mortality risk (RR = 0.61; 95% CI = 0.46–0.80) compared with those who did not change their physical activity level or were physically inactive/insufficiently active before diagnosis [35]. As of now, effects of physical exercise interventions on early or late cardiovascular endpoints in cancer survivors have not been published, although preclinical evidence demonstrates that exercise and caloric restriction can independently reduce anthracycline-related injury to the heart [61].

In an Australian intervention study, twenty-eight patients with early-stage breast cancer undergoing anthracycline chemotherapy were able to choose between exercise training or standard care [62]. In the intervention group, no differences were seen in LVEF, but decreases in V·O2 peak (representing peak aerobic fitness) during chemotherapy were attenuated, and functional status was preserved, compared to control. Some indirect evidence that exercise might preserve cardiovascular status in cancer patients comes from a prospective study in colon cancer survivors reporting that physical exercise caused lower insulin levels and insulin resistance, compared to standard of care [63]. A prospective study in breast cancer patients revealed that an exercise-based intervention programme protected from treatment-induced tachycardia and hypotension, but was only a temporary effect during the intervention [64].

Even though exercise or the initiation of such, after a cancer diagnosis may decrease risks for late cardiovascular disease, there is still lack of knowledge in among health professionals, that may well be due to some considerable heterogeneity and risk for bias in published studies, but also the various approaches that can be used and confirm the safety of exercise interventions [65]. In a recently updated evidence-based guideline, it is recommended that women with breast cancer follow the recommendations of 150 min of aerobic activity per week and strength exercise 2–3 times a week [66].

*Highest LOE for physical exercise and CVD endpoints: 5 (rationale for ongoing RCT’s)*.

### Strategies for prevention of CVD after cancer treatment completion

To our knowledge, no intervention studies have been performed that studied the role of strategies to prevent development of CVD after completion of oncological treatments. The guidelines from ASCO and ESMO on prevention of cardiac dysfunction related to oncological treatments recommend that clinicians complete a careful history and physical examination in survivors of cancer previously treated with potentially cardiotoxic therapies, and additional cardiac investigations in case of clinical signs and symptoms of heart failure [26, 27]. These guidelines also advocate regular evaluation and management of traditional cardiovascular risk factors and obesity in patients previously treated with cardiotoxic cancer therapies. A so-called ‘heart-healthy’ lifestyle, including advice on the importance of diet and exercise, should be discussed as part of long-term follow-up care. This is a recommendation based on intermediate quality evidence. It is not clear how frequently such evaluations should be performed. However, the ASCO and ESMO guidelines recommend an individualised approach based on patient- and treatment characteristics as well as risk factors for the development of treatment-related CVD. Predictive biomarkers, e.g. serum levels of troponins or natriuretic peptides, that identify patients increased risk for future CVD are needed to individualise follow-up and treatment schemes for cardiovascular risk management in cancer survivors.

The need for taking cancer treatments into account in patients with CVD, and the increased risk for CVD in cancer survivors, was also recently addressed in a study and accompanying editorial in Lancet [67, 68]. It is, however unclear whether target values, e.g. lipids and blood pressure should be equal or perhaps more stringent in cancer survivors than the general population, especially regarding primary prevention.

*Highest LOE for strategies for CVD prevention in cancer survivors: 5*.

### Ongoing intervention trials aiming to decrease CVD in relation to cancer treatments

At clinicaltrials.gov, we identified 17 actively recruiting prospective trials in adult cancer patients; almost all concern patients treated with anthracyclines and (in part) anti-HER2 agents as well (Table 4). Five studies are offering a physical exercise intervention, with training supervised by experts, whereas nine concern an intervention with medication from different classes. Interestingly, two studies are investigating the role of ivabradine. This is a class of drugs that, to our knowledge, has not yet been investigated in a cancer patient population with the aim to decrease treatment-related cardiac toxicity. It belongs to a class of medication called hyperpolarization-activated cyclic nucleotide-gated channel blockers, and the mechanism of action is that it slows the heart rate and thereby increases diastolic perfusion time and myocardial perfusion. Almost all studies (15 out of 17) have cardiac function assessed by ultrasound or cardiac MRI, either LVEF or global strain, as primary endpoint.

**Table 4:** Ongoing intervention studies aiming to reduce cardiovascular disease in cancer patients; only actively recruiting studies are mentioned (accessed 2021-03-22)

ClinicalTrials.gov ID	Population	Intervention	Primary endpoint	Estimated enrollment
NCT03964142	BC pts. treatment with anthracyclines and / or anti-HER-2 antibodies	comprehensive cardiac rehabilitation program including supervised exercise training	Change in left ventricular systolic function quantified by left ventricular ejection fraction and global longitudinal strain by transthoracic echocardiography	340
NCT04476576	hemato-oncological diseases that initiate chemotherapy	Aerobic exercise Vs Flexibility exercise	Change in systolic longitudinal strain (exercise of flexibility and balance). [Time Frame: 6 months]	38
NCT03850171	Pts with breast cancer or lymphoma scheduled for anthracycline-based chemotherapy	Exercise Training	Changes from baseline in left ventricular (LV) global longitudinal strain (GLS) [Time Frame: week 13]	120
NCT03787966	BC pts. scheduled for surgery and adjuvant chemotherapy	Exercise training before or before and after medical treatment	Left ventricular ejection fraction [Time Frame: Participants will be followed over 12 months]	100
NCT03711110	Elderly pts. Colon Cancer, Breast Cancer, Lymphoma, Chronic Lymphoma Leukemia, Multiple Myelom	Intensive cardiovascular monitoring	All-cause mortality [Time Frame: Two (mid-term analysis) and 5 years of follow-up]	514
NCT03186404	Pts with BC, lymphoma, leukemia or sarcoma scheduled for anthracycline-based chemotherapy	Atorvastatin	Cardiac MRI measured LVEF within 4 weeks of anthracycline completion	112
NCT04023110	breast Cancer Patients Treated With Doxorubicin and/or Trastuzumab	Carvedilol	Left Ventricular Ejection Fraction (LVEF) [Time Frame: up to 24 months	110
NCT03265574	Pts with BC and lymphoma treated with anthracyclines	Enalapril	Cardiac troponin T release [Time Frame: One month after last dose of anthracycline	170
NCT03650205	cancer pts. treated with anthracyclines	Ivabradine	Ventricular function [Time Frame: 365 days after randomization] Reduction in global longitudinal strain of at least 10% (GLS)	160
NCT04030546	cancer pts. treated with anthracyclines	Ivabradine	Change in left venticular dysfunction by global longitudinal strain (GLS). [Time Frame: 1, 3 and 6 months] Change in global longitudinal strain (GLS) at least by 3%.	128
NCT04429633	breast cancer patients treated with adjuvant trastuzumab.	Candesartan	Left ventricular ejection fraction (LVEF) [Time Frame: at months 3,6,9,12,18]	136
NCT02818517	Two cohorts, one prospective group will include all oncologic patients who will be evaluated in the cardio-oncology clinic	ACE inhibitors or beta blockers	ECho-global strain [Time Frame: 2 years	1000
NCT04092309	Pts undergoing Hematopoietic Stem Cell Transplantation	Sacubitril-Valsartan	Left Ventricular Function, Global Longitudinal Strain, arterial stiffness, glycocalyx thickness [Time Frame: 2 years]	90
NCT02943590	Pts with newly diagnosed NHL and HL, anthracycline-based therapy	Atorvastatin	Left ventricular Ejection Fraction (LVEF) [Time Frame: 12 months]	270
NCT04632407	BC pts. during adjuvant chemotherapy	Dietary Supplement: Flax “milk”Dietary Supplement: Oat fibre “milk”	Left ventricular ejection fraction (LVEF) change [Time Frame: 1 year]	60
NCT03934905	BC pts. neoadjuvant treatment incl doxorubicin	sulforaphane	Change in cardiac function after DOX therapy with or without sulforaphane through diagnostic studies [Time Frame: At baseline and 1 year from baseline assessment.]	70
NCT04361240	Breast cancer radiotherapy	Radiation: Proton vs Photon Radiation	Change in echocardiography derived LVEF from baseline	155

## Conclusion

Our literature review identified studies with moderate to good strength and up to now limited evidence to recommend primary preventive strategies in unselected patients treated with potentially cardiotoxic oncologic therapies (Table 5). Some predictive models exist, but all lack methodological robustness to be implemented in clinical use.

**Table 5:** Levels of Evidence for methods of prevention of cardiovascular disease related to cancer treatment

Timing	Intervention	Effect	Level of Evidence	Reference
**Pre-treatment**	Use of predictive models for future CVD	Risk groups to predict rate of cardiac events during 3 year follow-up after curative (HER2+) BC treatment	2b	[15]
**During treatment**	ACEi/ARB	Mean difference in delta LVEF −2.36% in pts. with early HER2+/− BC	1a	[28]
	Beta-blocker	2,6% lower incidence of LVEF < 50% compared to placebo, NNT 38	1a	[29]
	Statins	No difference in LVEF < 50%	1b	[39]
	Aspirin/anticoagulants	n.a.	n.a.	
	Other drugs; dexrazoxane	Reduces risk of CHF and cardiac events	1a	[40]
	Other drugs; sildenafil	No difference LVEF-declines	1b	[42]
	Smoking cessation	Circumstantial: smokers increased reisk for cardiac toxicity related to radiotherapy	n.a.	
	Weight loss	Circumstantial: reduction non-BC mortality in BC survivors	n.a.	
	Physical exercise	Hypothesis generating for ongoing RCT’s	5	
**Post treatment**	Primary prevention	Indirect evidence from general population	n.a.	

*Abbreviations*: *CVD* cardiovascular disease, *HER2* Human Epidermal growth Receptor 2, *ACEi* ACE-inhibitor, *ARB* angiotensin receptor blocker, *LVEF* left ventricular ejection fraction, *n.a.* not available, *BC* breast cancer, *RCT’s* randomized controlled trials, *CHF* clinical heart failrure

In current clinical practice, based on the data reviewed here, we recommend refraining from offering patients with a cancer diagnosis primary prevention with medication concomitantly with a potential cardiotoxic treatment. Meta-analyses support the role of beta-blockers or ACEI/ARB, although the magnitude of effect is very modest in unselected patients, and in our opinion too little to justify the potential side-effects, interactions with oncological therapies and costs.

At the same time, based mainly on data from retrospective analyses, we do emphasize that all clinicians should advocate the beneficial role of life-style optimization by means of a heart-healthy diet and meeting recommendations for level of physical exercise in all patients scheduled for such treatments. Although there is clearly less evidence to support such interventions in terms of cardioprotective capacities, is there ample evidence on other clinically relevant outcomes to pursue healthy life-style behaviour in our patients. Currenlty ongoing trials will hopefully reveal whether such interventions will be effective in decreasing cardiovascular complications of oncological treatments.

In the future, a patient at increased risk for treatment-related CVD (both acute and late onset) should ideally be identified through an objective, practically usable and externally validated baseline risk estimation score (Fig. 1). Such a risk score should be easily accessible and based on patient- and treatment-related factors, and in addition, biomarkers for subclinical cardiovascular damage (at baseline or during treatment) could be included. Patients that are, based on such a risk assessment, deemed at highest risk would be ideal candidates to include in an intervention trial that aims to decrease cancer treatment-related cardiovascular toxicity in order to improve cancer survivorship by optimizing outcomes whilst minimizing the risk for unwanted side-effects.

## Data Availability

Not applicable
